# Combination of Selective Etching and Impregnation toward Hollow Mesoporous Bioactive Glass Nanoparticles

**DOI:** 10.3390/nano11071846

**Published:** 2021-07-16

**Authors:** Nurshen Mutlu, Ana Maria Beltrán, Qaisar Nawaz, Martin Michálek, Aldo R. Boccaccini, Kai Zheng

**Affiliations:** 1 Institute of Biomaterials, Department of Material Science and Engineering, University of Erlangen-Nuremberg, 91058 Erlangen, Germany; nurshen.mutlu@fau.de (N.M.); qaisar.nawaz@fau.de (Q.N.); 2 FunGlass, Department of Biomaterials, Alexander Dubček University of Trenčín, Študentská 2, 911 50 Trenčín, Slovakia; martin.michalek@tnuni.sk; 3 Departamento de Ingeniería y Ciencia de los Materiales y del Transporte, Escuela Politécnica Superior, Universidad de Sevilla, 41011 Seville, Spain; abeltran3@us.es

**Keywords:** bioactive glasses, hollow mesoporous structure, alkaline etching, impregnation, bone regeneratio

## Abstract

In this study, binary SiO_2_-CaO hollow mesoporous bioactive glass nanoparticles (HMBGNs) are prepared by combing selective etching and impregnation strategies. Spherical silica particles (SiO_2_ NPs) are used as hard cores to assemble cetyltrimethylammonium bromide (CTAB)/silica shells, which are later removed by selective etching to generate a hollow structure. After the removal of CTAB by calcination, the mesoporous shell of particles is formed. Calcium (Ca) is incorporated into the particles using impregnation by soaking the etched SiO_2_ NPs in calcium nitrate aqueous solution. The amount of incorporated Ca is tailorable by controlling the ratio of SiO_2_ NPs:calcium nitrate in the soaking solution. The produced HMBGNs are bioactive, as indicated by the rapid formation of hydroxyapatite on their surfaces after immersion in simulated body fluid. In a direct culture with MC3T3-E1 cells, HMBGNs were shown to exhibit concentration-dependent cytotoxicity and can stimulate osteogenic differentiation of MC3T3-E1 cells at concentrations of 1, 0.5, and 0.25 mg/mL. Our results indicate that the combination of selective etching and impregnation is a feasible approach to produce hierarchical HMBGNs. The produced hollow particles have potential in drug delivery and bone tissue regeneration applications, and should be further investigated in detailed in vitro and in vivo studies.

## 1. Introduction

Bioactive glass nanoparticles (BGNs) are emerging multifunctional (e.g., osteogenic, angiogenic, antibacterial) materials for a variety of biomedical applications, including bone regeneration, wound healing, and tumor treatment [1,2]. Their composition and pore structure can be tailored to achieve enhanced therapeutic effects for specific applications, for example, ion-doped mesoporous BGNs (MBGNs) have been developed for co-delivery of drugs and biologically active ions for bone repair and regeneration [3]. Recently, hollow MBGNs (HMBGNs) are gaining increasing attention in therapeutic and diagnostic applications due to their large specific surface area and pore volume leading to enhanced bioactivity, biological response, and drug loading capacity [4,5,6,7]. Compared to non-hollow MBGNs, HMBGNs possess a unique internal reservoir space that enables them to store a larger amount of drugs and to achieve a more controlled release [8]. HMBGNs also exhibit advantages over other hollow mesoporous particle systems due to their capability of delivering biologically active ions. They are; thus, considered one of the most promising platforms able to synergistically release ions and drugs for disease treatment and tissue regeneration [3].

Synthesis strategies toward mesoporous particles have been well established by using pore-forming templates (e.g., copolymers, cationic surfactants) in sol–gel processing to guide mesopore formation [9]. Sol–gel-based templating methods have also been used to synthesize hollow mesoporous particles, in which the key is to generate the void inside the particles. To this end, either soft templates or hard templates can be applied as cores to assembly mesoporous shells [10,11]. Various soft templates as cores have been used to assist the synthesis of HMBGNs, such as microemulsion and micelles [5,12,13,14]. However, the use of soft templates may cause inhomogeneity in size and shape as well as aggregation of resulting particles, especially when metal precursors (e.g., metal salts) for introducing metal ions are added in sol–gel processes [10,11]. Alternatively, hard templates usually lead to hollow particles with homogenous particle size and shape due to the stability and uniform morphology of hard templates [15]. In this strategy, hard templates are employed as cores to assemble hybrid silicate species/micelle shells on the surface. After the removal of templates (cores and micelles), hollow mesoporous particles are formed. The diameter of hollow mesoporous particles can be controlled by tailoring the size of core templates.

Spherical Stöber-derived silica particles are favorable templates to produce hollow mesoporous particles due to their high dispersibility, homogeneous spherical shape, and uniform and tunable particle size [10]. Silica particles can be conveniently removed by etching in hot water or alkaline solution (e.g., NaOH, Na_2_CO_3_) [10]. However, the etching process is not friendly to the synthesis of HMBGNs, as the process can also remove the incorporated metal ions (typically Ca ions). Therefore, the Stöber method-derived silica particles are rarely used as the template to produce HMBGNs, though other micro-sized hard templates (e.g., pollen grains, polystyrene particles) have been applied to produce hollow BG particles [16,17].

**Figure 1:**
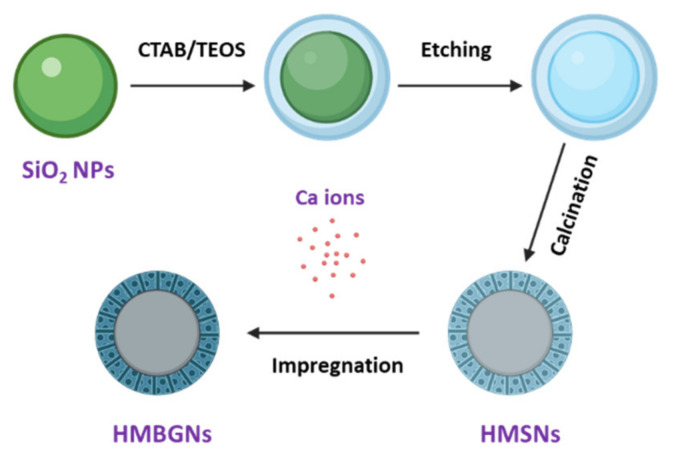
Schematic illustration of the synthesis process of HMBGNs.

Recently, our group has introduced a post impregnation strategy to produce MBGNs in order to avoid particle aggregation and formation of metal or metal oxide nanoparticles as side products in sol–gel processes [18]. In the present study, we successfully produced binary SiO_2_-CaO HMBGNs by using the Stöber-derived silica particles as the hard template and combing etching and impregnation strategies. Figure 1 shows a schematic illustration of the HMBGN synthesis process. In this method, homogenous, uniform silica nanoparticles (~400 nm) were employed as the cores to assemble mesoporous shells. The hollow structure was obtained by etching the hard core using alkaline Na_2_CO_3_ solution. Calcium (Ca) was introduced into the hollow mesoporous particles using an impregnation method. The amount of incorporated Ca could be controlled by tuning the ratio between particles and impregnation solution (calcium nitrate aqueous solution). The morphology, structure, in-vitro apatite forming ability, cytotoxicity, and osteogenic potential of the produced HMBGNs were evaluated.

## 2. Materials and Methods

### 2.1. Synthesis of Silica Nanoparticles

All chemicals used in this study were purchased from Sigma-Aldrich (Darmstadt, Germany) without further purification. Silica nanoparticles (SiO_2_ NPs) were synthesized using a modified Stöber method as described in our previous study [19]. In a typical synthesis, solution A was prepared by adding 3 mL of tetraethyl orthosilicate (TEOS) in ethanol (25 mL), while solution B was prepared by mixing ammonium hydroxide solution (4.5 mL, 28 wt%), ethanol (8.2 mL), and deionized water (12.4 mL). Next, solution A was poured into solution B under continuous stirring. The reaction continued for 2 h to form colloidal SiO_2_ NPs with particle size ~400 nm. The SiO_2_ NPs were then collected by centrifugation and washed once with water and once with ethanol. The as-synthesized SiO_2_ NPs were dried at 60 °C for further experiments. The size of SiO_2_ NPs could be adjusted by tuning concentrations of used water, ethanol, and ammonia [19]. However, in this study, only 400 nm SiO_2_ NPs were used as the hard template.

### 2.2. Synthesis of Hollow Mesoporous Silica Nanoparticles by Etching

Hollow mesoporous silica nanoparticles (HMSNs) were fabricated using a selective etching strategy as described in a previous study [10]. In brief, 800 mg of the dried SiO_2_ NPs were dispersed in deionized water (80 mL). 800 mg of cetrimonium bromide (CTAB), 160 mL of deionized water, 80 mL of ethanol, and 2.2 mL of ammonium hydroxide (28%) were then sequentially added in the suspension. After 30 min of reaction under continuous stirring, 2 mL of TEOS was added and left reaction for further 6 h. Afterwards, the particles were collected by centrifugation and washed twice using deionized water and then dispersed in 160 mL of deionized water. After that, 1.7 g of Na_2_CO_3_ was added to the suspension under stirring at 80 °C for 24 h to etch the core of SiO_2_ NPs. Subsequently, the etched particles were collected by centrifugation and washed twice with deionized water and once with ethanol. The collected particles were used for further experiments

### 2.3. Incorporation of Calcium by Impregnation

In order to obtain bioactive particles, calcium, the essential element for BGs, was incorporated into the etched particles using an impregnation strategy as reported in the literature [18,20]. In a typical process, 300 mg of etched particles were soaked in 20 mL of calcium nitrate aqueous solution for 1 h under stirring at room temperature (~25 °C). After soaking for 1 h, the particles were centrifuged and washed twice with deionized water. The collected particles were dried at 60 °C overnight before calcination at 700 °C for 3 h. The molar ratios of Ca/Si in the soaking solution were set as 1:1 and 2:1 and the obtained particles were designated as 1CaHMBGNs and 2CaHMBGNs, respectively. Hollow mesoporous silica nanoparticles without the impregnation treatment were used as the control group (HMSNs).

### 2.4. Characterization of HMBGNs

The morphology and microstructure of HMBGNs were characterized using field emission scanning electron microscope (FE-SEM; Auriga, Zeiss, Oberkochen, Germany) and probe Cs-corrected scanning transmission electron microscopy (STEM, TALOS F200S, FEI, Eindhoven, The Netherlands). The composition of nanoparticles was analyzed using energy dispersive spectroscopy (EDS, X-Max^N^ Oxford Instruments, Abingdon, UK) at an accelerating voltage of 15 kV and a working distance of 6 mm during SEM imaging. Fourier-transform infrared spectroscopy (FTIR) was performed on the samples using a Nicolet 6700 FTIR spectrophotometer (Thermo Scientific, Waltham MA, USA) in transmission mode under ambient conditions. For the measurement, the samples were mixed with KBr at a ratio of 1:100 (*w*/*w*) and made into pellets. Spectra were collected with a resolution of 4 cm^−1^. Powder X-ray diffraction (XRD) was performed using a Philips X’pert diffractometer (Philips, Eindhoven, The Netherlands) in the 2Ɵ range of 10–80° with Cu Kα radiation. A step size of 0.020° with a dwelling time of 1 s per step was applied. The Brunauer–Emmett–Teller (BET) specific surface area and pore structure of the samples were determined by using nitrogen adsorption-desorption analysis on ASAP2020 (Micromeritics, Atlanta, GA, USA). The samples were degassed at 150 °C for 6 h before the measurement.

### 2.5. In-Vitro Hydroxyapatite Formation

In-vitro bioactivity of HMBGNs was accessed by soaking the particles in simulated body fluid (SBF) to observe the formation of hydroxyapatite (HA) according to the protocol proposed by Kokubo and Takadama [21]. Briefly, HMNGNs were soaked in SBF at a concentration of 1 mg/mL and kept in an incubator (KS 4000i control, IKA, Staufen, Germany) at 37 °C, 90 rpm for 7 days. At the predetermined time point, the samples were collected by centrifugation and washed with DI water and acetone (100%, VWR Chemicals, Radnor, TN, USA). After drying, the samples were analyzed by FTIR, XRD, and SEM to observe the formation of HA. The characterization procedure was the same as described above.

### 2.6. Cell Viability

Mouse preosteoblast cells (MC3T3-E1), derived from mouse calvaria (Sigma-Aldrich, Taufkirchen, Germany), were cultured in α-MEM (Minimum Essential Medium Eagle—alpha modification) (Sigma-Aldrich, Taufkirchen, Germany) supplemented with 10% fetal serum (FBS, Sigma-Aldrich, Taufkirchen, Germany), 1% penicillin-streptomycin (PS, Life Technology, Darmstadt, Germany) solution and 1 vol% L-Glutamine (Life Technology, Darmstadt, Germany). Conditioned cell culture media were prepared by dispersing sterilized glass particles (sterilization by heating at 160 °C for 2 h) in α-MEM at concentrations of 1, 0.5, 0.25, and 0.1 mg/mL. The culture medium without samples was used as a control. Cell suspension of 25,000 cells/per well was seeded and incubated at 37 °C for 24 h in a CO_2_ incubator in the atmosphere of 95% air and 5% CO_2_. Then, the media were replaced with the conditioned α-MEM. After culture for 3 days (3D), cell viability was evaluated. Briefly, the culture medium was removed and washed with PBS three times to remove unattached cells and particles and incubated with 1% WST-8 reagent (CCK-8, Taufkirchen, Sigma-Aldrich) in phenol red-free α-MEM for 3 h at 37 °C/5% CO_2_. The solution was transferred into a 96-well plate to measure absorbance at 450 nm using a microplate Elisa reader. All experiments were made in triplicate. The viability of the MC3T3-E1 cells was calculated according to the equation:

$$Cell\;viability\;\left(\%\right)=\frac{\left(Absorbance\;of\;sample-Absorbance\;of\;blank\right)}{\left(Absorbance\;of\;positive\;control-Absorbance\;of\;blank\right)}.$$

### 2.7. Alkaline Phosphatase Activity Assay

In the alkaline phosphatase activity (ALP) test, osteogenic differentiation media (osMEM) was prepared by supplementing a regular culture medium with 50 µg/mL ascorbic acid, 10 mM β-glycerophosphate, and 10 nM dexamethasone. HMBGNs were dispersed in osMEM at concentrations of 0.1, 0.25, 0.5, and 1 mg/mL. The control group was exposed to osMEM without glass particles. The media was changed two times per week. To quantify alkaline phosphate (ALP) activity, cells were seeded into 24-well plates at 25,000/per well and cultured for 14D with the particles at concentrations mentioned above in osMEM. The cells were rinsed with PBS three times, followed by treating lysis buffer for 30 min at 37 °C, and stored at −20 °C until measurement. ALP expression was measured by an assay based on the change in absorbance of p-nitrophenylphosphate (pNpp) as it is enzymatically cleaved by ALP to p-nitrophenol (pNp). The lysates were centrifuged for 5 min at 2000 rpm. Afterwards, 250 µL of the supernatant was transferred to a cuvette and 100 µL of 2.36 mg·mL^−1^ pNpp solution was added and incubated for 5 h. The reaction stopped by adding 650 µL 1M NaOH solution and the absorbance was measured at 405 by a UV–Vis spectrometer (Specord 250, Analytikjena, Jena, Germany).

### 2.8. Statistical Analysis

Statistical analysis was performed by one-way ANOVA and Tukey’s test using the software Origin 2017 (OriginLab, Northampton, MA, USA). The probability (*p*) values *p* < 0.05 were considered statistically significant. The results were expressed as mean ± standard deviation (SD).

## 3. Results and Discussion

### 3.1. Synthesis of Hollow Mesoporous Bioactive Glass Particles

**Figure 2:**
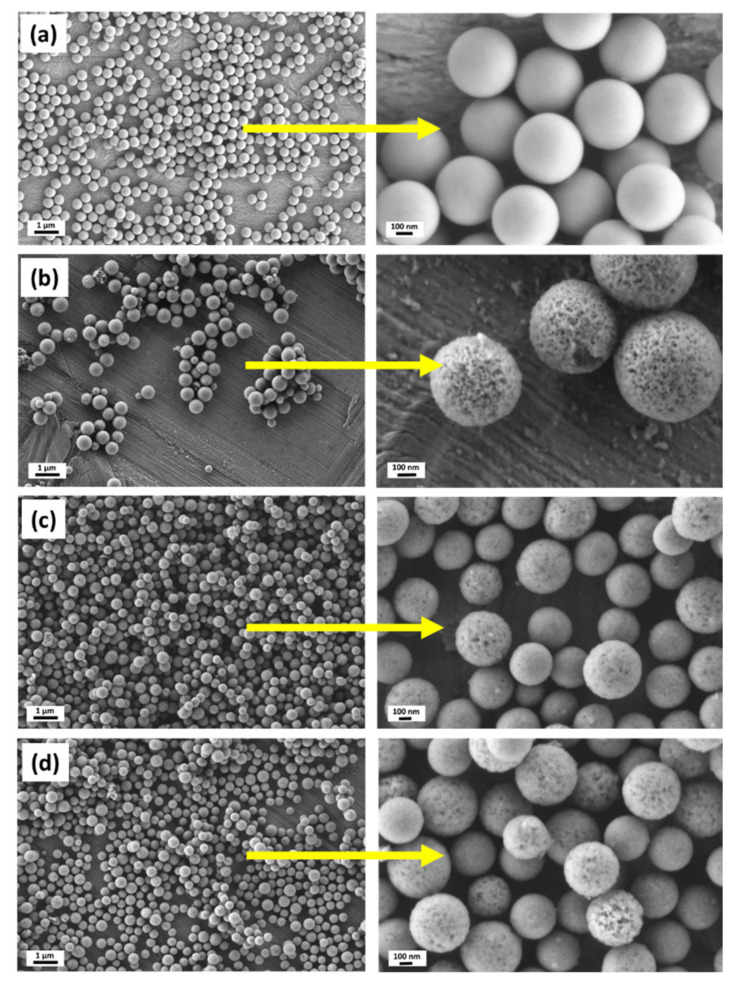
SEM images of (**a**) SiO_2_ NPs; (**b**) HMSNs; (**c**) 1CaHMBGNs; and (**d**) 2CaHMBGNs.

**Figure 3:**
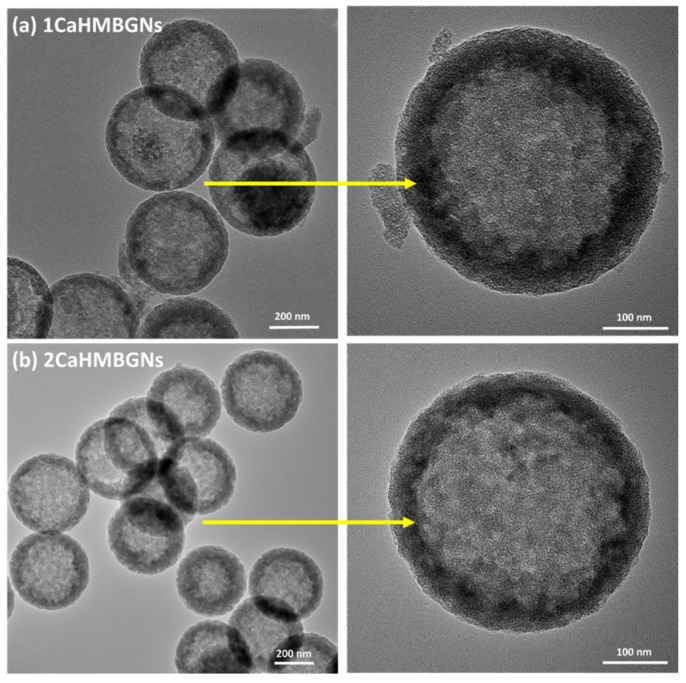
TEM images of hollow mesoporous bioactive glass particles: (**a**) 1CaHMBGNs and (**b**) 2CaHMBGNs.

In this study, we used a two-step approach to synthesize binary SiO_2_-CaO HMBGNs. Figure 1 shows the schematic illustration of the synthesis process. SiO_2_ NPs were firstly synthesized using a modified Stöber method. The particles were monodispersed and exhibited uniform spherical shape with a particle size of ~400 nm, as shown in SEM images (Figure 2a), which is a typical morphology of nanoparticles synthesized by using the Stöber method. The particle size of SiO_2_ NPs can be controlled by tuning processing parameters (e.g., concentrations of catalysts and precursors, water/ethanol ratio) [19]. Here we selected SiO_2_ NPs with a size of 400 nm as the hard template. Figure 2b shows SEM images of HMSNs that were generated by a selective etching method. Nanopores can be observed on the surface of the etched particles. The size of hollow particles was approximately 400 nm, similar to the size of SiO_2_ hard templates. After the synthesis of HMSNs, the essential element Ca for BGs was incorporated into the particles using a post impregnation method. Figure 2c,d shows SEM images of 1CaHMBGNs and 2CaHMBGNs. Their porous structure and particle size were not significantly affected by the impregnation process in comparison to HMSNs. Also, the dispersibility of particles seems not to be reduced after the incorporation of Ca. Figure 3 shows representative TEM images of 1CaHMBGNs and 2CaHMBGNs. As can be seen, both particle types exhibit hollow structure with mesoporous shells. The thickness of mesoporous shells appears to be in the range of 20 to 30 nm, which could be tailored by varying the added amount of CTAB and TEOS in the synthesis process [22].

In this work, SiO_2_ NPs were used as the hard template to fabricate hollow particles. After the assembly of CTAB and silicate species, the silica templates were etched under alkaline conditions based on the selective etching mechanism [22,23]. The hybrid silicate shells were formed by the hydrolysis/condensation of the CTAB-TEOS mixture. They exhibited a lower amount of Si-OH groups than silica cores and; therefore, possessed a higher degree of condensation of the Si-O-Si network, leading to a more stable shell than the core under Na_2_CO_3_ aqueous solution [23]. Therefore, during the etching process, the outer CTAB/silicate shell could remain intact while the inner SiO_2_ core was readily etched. After the removal of CTAB by calcination, hollow mesoporous particles were obtained (Figure 3). However, it is still challenging to incorporate metal ions into this type of hollow particle in one-step synthesis, as the etching process would remove the incorporated metal ions. Alternatively, we selected a post impregnation method to incorporate Ca into the hollow particles. This approach has been used to include metal ions into nanoparticles [18,20]. Our results showed that the post impregnation did not affect the hollow mesoporous structure of the particles (Figure 3), which was consistent with the findings in previous studies [18,20].

**Figure 4:**
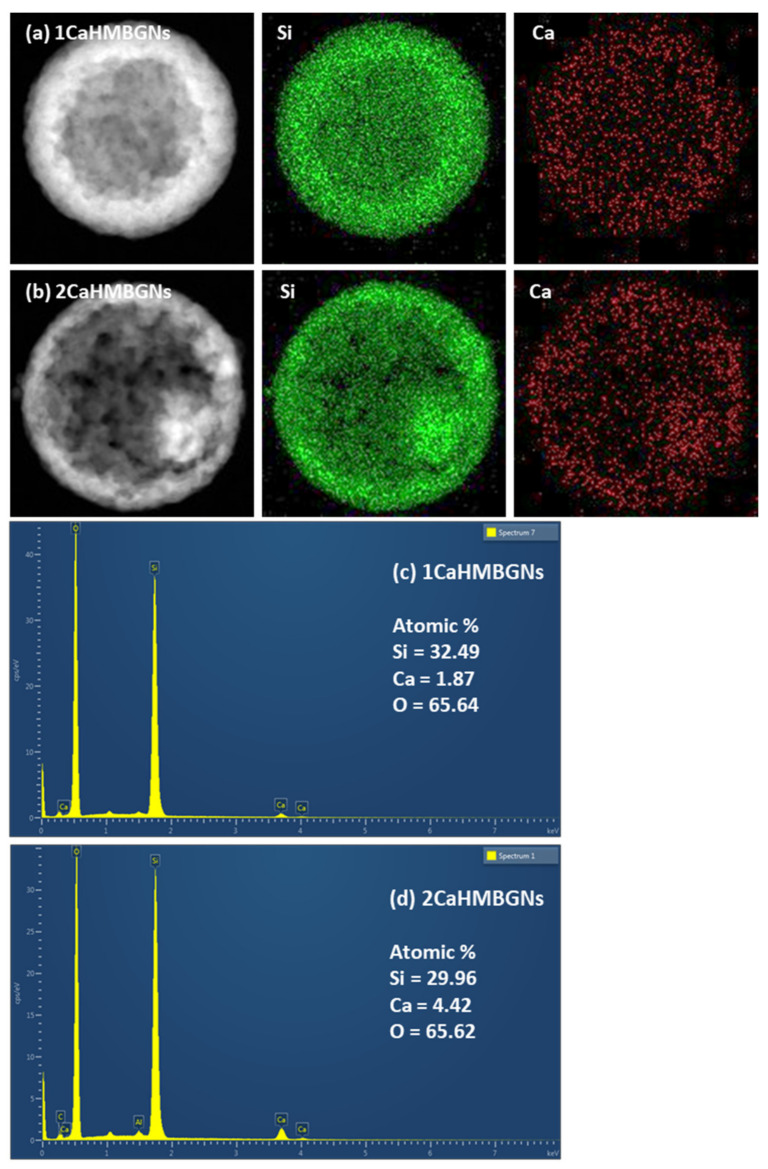
EDS mapping of (**a**) 1CaHMBGNs and (**b**) 2CaHMBGNs; and EDS spectra of (**c**) 1CaHMBGNs and (**d**) 2CaHMBGNs. The size of particles shown in (a) and (b) is ~400 nm.

We further evaluated the amount of incorporated Ca in the hollow particles. Figure 4a,b shows EDS mapping of 1CaHMBGNs and 2CaHMBGNs. The image reveals that Ca was successfully incorporated into HMBGNs and homogeneously distributed throughout the particles. The amount of calcium precursor (calcium nitrate in this study) used for impregnation did not affect the particle size and pore structure of HMBGNs (Figure 3). This finding agrees with the result in our previous study showing that the concentration of metal ion precursor solution did not affect the particle morphology [18]. However, the amount of incorporated Ca was indeed affected by the concentration of Ca precursor. Figure 4c,d show EDS spectra and the obtained atomic concentrations of Si and Ca in HMBGNs. As seen in the spectra, both 1CaHMBGNs and 2CaHMBGNs contained Si and Ca, while their concentrations of CaO were calculated to be ~5.4 and 12.9 mol%, respectively. The results indicated that a higher concentration of impregnation solution could increase the amount of Ca incorporated in the hollow particles. This finding indicates that the incorporated amount of Ca in HMBGNs can be controlled in the impregnation approach. In addition to tuning the concentration of impregnation solution, the incorporated amount of metal ions can also be controlled by adjusting the impregnation temperature. However, an elevated temperature can increase the risk of side product formation (e.g., metal or metal oxide nanoparticles) [18].

In this study, we successfully used SiO_2_ NPs as the hard template, which ensured the homogenous size and shape of resulting HMBGNs (Figure 2). A selective etching process was used to generate the hollow structure (Figure 3). Moreover, to avoid the loss of Ca ions during the etching process, we applied a post impregnation strategy to incorporate Ca (Figure 4). The presented results indicate that the combination of selective etching and impregnation is an effective strategy for synthesizing HMBGNs. It is expected that other metal ions can also be incorporated HMBGNs using this strategy.

### 3.2. Structural Characterization and In-Vitro Apatite Forming Ability of HMBGNs

**Figure 5:**
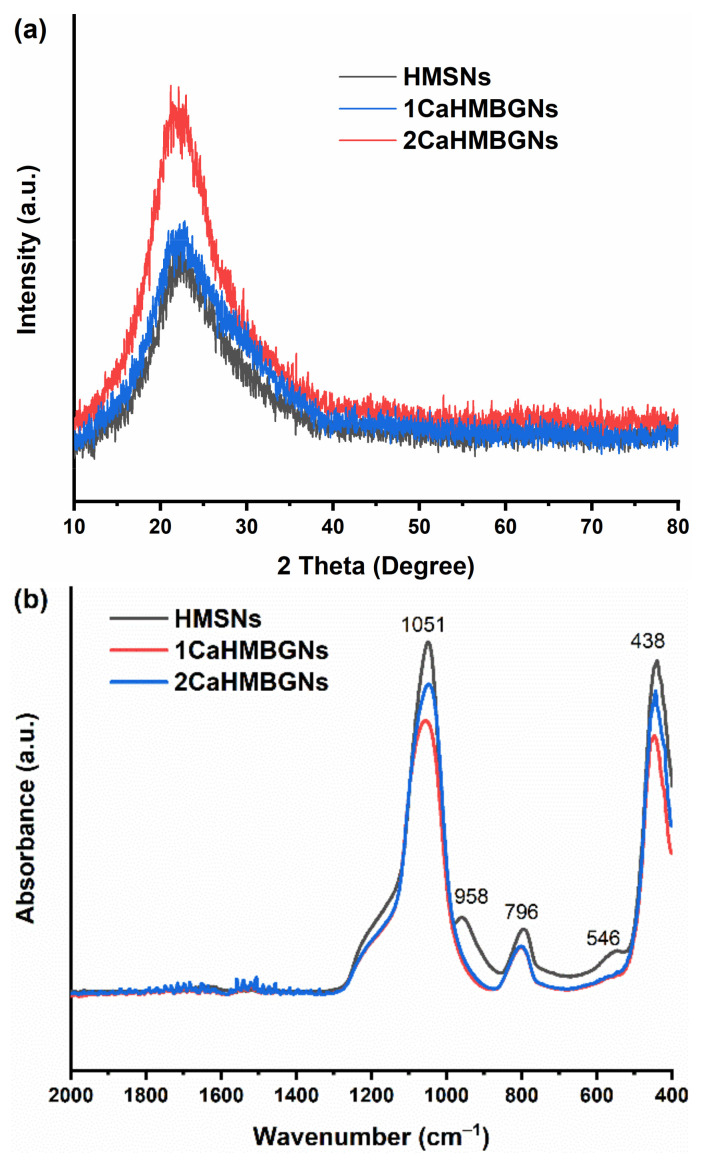
XRD patterns (**a**) and FTIR spectra (**b**) of HMSNs, 1CaHMBGNs, and 2CaHMBGNs.

We further evaluated the structure of HMBGNs. Figure 5a shows the XRD patterns of the hollow particles before and after impregnation. HMSNs, 1CaMBGNs and 2CaMBGNs exhibited the typical diffraction pattern of amorphous silicate materials, in which only a broad band located between 2*θ* = 15° to 35° corresponding to amorphous silicate could be observed [24]. The incorporation of Ca and its amount did not affect the amorphous characteristic of HMBGNs. Figure 5b shows the FTIR spectra of HMSNs, 1CaHMBGNs, and 2CaHMBGNs. All hollow particles exhibited characteristic bands corresponding to silicate glasses. Two bands located at ∼438 and 796 cm^−1^ could be assigned to Si-O-Si bending and symmetric stretching vibrations, respectively. The band located at 1051 cm^−1^ could be assigned to Si-O-(non-bridging bonds) or asymmetric Si-O-Si (bridging bonds) vibrations [25,26]. After incorporation of Ca, the band located at 960 cm^−1^ assigned to Si-OH symmetric stretching vibration was not observed [25], which suggested the interaction between Ca and Si-OH leading to the formation of Si-O-Ca.

**Figure 6:**
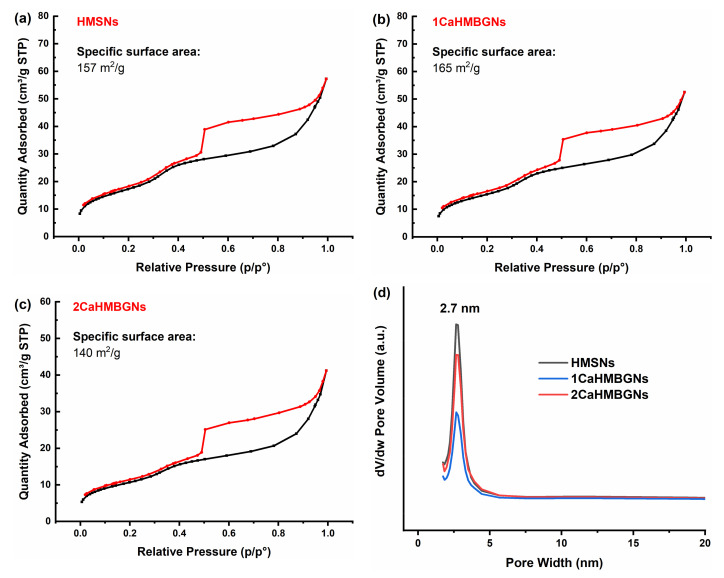
Nitrogen sorption isotherms of (**a**) HMSNs, (**b**) 1CaHMBGNs, and (**c**) 2CaHMBGNs, and their (**d**) pore size distribution.

Figure 6 shows nitrogen sorption isotherms of the calcinated HMSNs, 1CaMBGNs, and 2CaHMBGNs. All particles exhibited a type IV isotherm, a characteristic nitrogen sorption isotherm of hollow mesoporous particles. They also had a large hysteresis loop in the P/P_0_ range of 0.5−1.0, probably resulting from the capillary condensation in the mesoporous shell [22,27]. All hollow mesoporous particles possessed a pore size of ~2.7 nm, a typical mesopore size using CTAB as the pore-forming template. In addition to TEM images, nitrogen sorption results also confirmed the mesoporous structure of these hollow particles. After Ca incorporation, the nitrogen sorption isotherms and pore size of particles were not affected, indicating that the post impregnation process did not affect the mesoporous structure of the particles. HMSNs, 1CaMBGNs, and 2CaMBGNs also exhibited large BET surface area of ~157, ~165, and ~140 m^2^/g, respectively. Due to the relatively large particle size of these particles (~400 nm), their specific surface area was lower than that of hollow mesoporous particles (~440 m^2^/g) with a smaller size (~100 nm) reported in the literature [27]. Nevertheless, the large specific surface area of 1CaHMBGNs and 2CaHMBGNs is favorable for their interaction with biomolecules beneficial for their applications in regenerative medicine.

**Figure 7:**
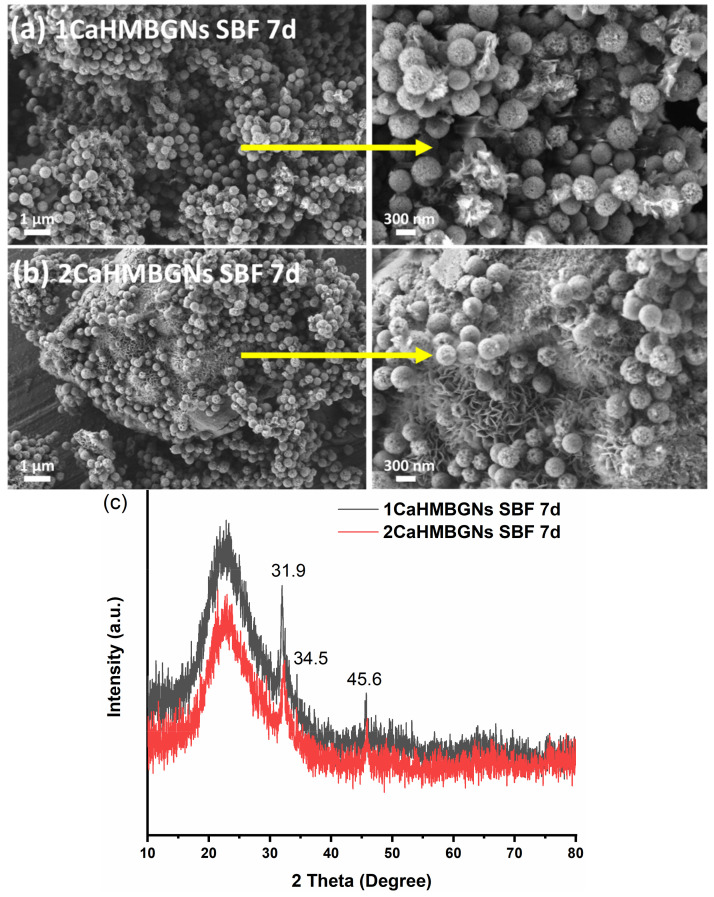
In-vitro hydroxyapatite formation of HMBGNs; (**a**,**b**) SEM images of 1CaHMBGNs and 2CaHMBGNs after immersion in SBF for seven days; (**c**) XRD patterns of HMBGNs after immersion in SBF for seven days. The marked peaks at 2*θ* = 31.9°, 34.5°, and 45.6° corresponded to HA peaks (JCPD 84-1998) [24].

We then evaluated the in-vitro hydroxyapatite (HA) forming ability of HMBGNs by immersing the particles in SBF. Figure 7a,b shows SEM images of 1CaHMBGNs and 2CaHMBGNs after immersion in SBF for seven days. Needle-like nanocrystals, which either formed clusters or adhered to the particles, can be observed in SEM images. The needle-like shape is the typical morphology of HA crystals formed on BG surface after immersion in SBF. The XRD results (Figure 7c) confirmed that the formed nanocrystals were HA as diffraction peaks located at approximately 2θ = 31.9°, 34.5°, and 45.6° attributed to HA crystals (JCPD 84-1998) can be observed in the XRD patterns of both HMBGNs [24]. It is well-known that BGs can bond with bone tissue through the formation of HA layer [28]. When incorporated into polymer matrices as rigid fillers, BGs can also release active ions (e.g., Ca ions), inducing HA formation on the matrices, which facilitates the bonding with bone tissue [3]. In this study, the produced HMBGNs could induce rapid HA formation in SBF (within seven days), indicating their favorable bioactivity beneficial for bone repair and regeneration.

### 3.3. In-Vitro Cytotoxicity and ALP Activity

**Figure 8:**
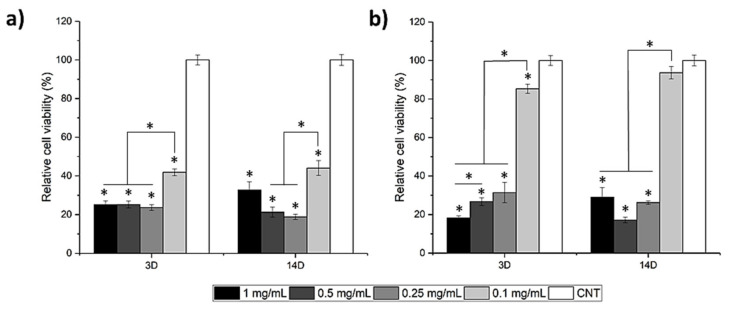
Relative cell viability of (**a**) 1CaHMBGNPs and (**b**) 2CaHMBGNPs at 1, 0.5, 0.25, and 0.1 mg/mL concentration after culture for three (3D) and 14 days (14D). Data are presented as mean ± SD and * indicates *p* < 0.05.

To evaluate the cytotoxicity of HMBGNs, we cultured the particles with MC3T3-E1 cells in a direct culture approach. Figure 8 shows the viability of MC3T3-E1 cells cultured with 1CaHMBGNs and 2CaHMBGNs for three and 14 days at concentrations of 1, 0.5, 0.25, and 0.1 mg/mL. It seems that the HMBGNs exhibited concentration-dependent cytotoxicity against MC3T3-E1 cells. At relatively high concentrations (1, 0.5, and 0.25 mg/mL) of particles used, both HMBGNs showed cytotoxicity toward MC3T3-E1 cells, as indicated by the low relative cell viability on both tested days. However, at a relatively low concentration of 0.1 mg/mL, 1CaHMBGNs and 2CaHMBGNs exhibited significantly higher cell viability on day three. Notably, the relative cell viability of 2CaHMBGNs group on day three was ~85%, indicating their non-cytotoxicity. After 14 days of culture, the relative cell viability of HMBGNs at 0.1 mg/mL increased, indicating the potential of these particles to promote cell proliferation in this direct cell culture.

BGs are generally considered as non-cytotoxic materials, though glass composition, particle concentration, and cell culture process can affect the viability of co-cultured cells [1,2]. Compared to the indirect cell culture method (dissolution products of materials are used in the cell culture), nanoparticles in the direct cell culture method usually induce higher cytotoxicity, as the particle size, shape, and surface chemistry can also influence cells [29]. In addition, in the direct cell culture method, nanoparticle uptake by cells or generation of reactive oxygen species (ROS) could occur, which may also induce cytotoxicity [30]. For example, He et al. [31] investigated the correlation between particle size of mesoporous silica nanoparticles (MSNs) and cytotoxicity. Their results showed that MSNs (190 and 420 nm) in a direct in-vitro culture method exhibited higher cytotoxicity than micron-sized particles (1200 nm) at concentrations from 10 to 480 µg/mL. In addition, their experimental results suggested that smaller MSNs were more easily endocytose, which also contributed to the higher cytotoxicity. It has also been reported that SiO_2_ NPs could induce oxidative stress and ROS when directly cultured with cells [32]. High levels of ROS may cause cell apoptosis and DNA damage [33]. In this study, we used a direct cell culture method to evaluate the cytotoxicity of HMBGNs, considering their potential application in drug delivery in which HMBGNs would be directly in contact with various cells. Our results showed that HMBGNs exhibited cytotoxicity against MC3T3-E1 cells at the relatively high concentrations of particles used, which is consistent with results in the literature [29,31]. At 0.1 mg/mL, 2CaHMBGNs showed lower cytotoxicity than 1CaHMBGNs. In addition, 2CaHMBGNs could also promote cell proliferation at 0.1 mg/mL. Given the similar morphology and surface chemistry between 1CaHMBGNs and 2CaHMBGNs, the higher amount of Ca in 2CaHMBGNs might contribute to these differences, as it has been reported that the incorporation of Ca in particles could positively affect the proliferation of osteoblasts and stem cells [34]. Our results indicate that the concentration of HMBGNs should be carefully selected for specific applications. When used as fillers in composites, a higher concentration of HMBGNs should not induce significant cytotoxicity in comparison to the direct use, since, in composites, HMBGNs act on cells indirectly.

**Figure 9:**
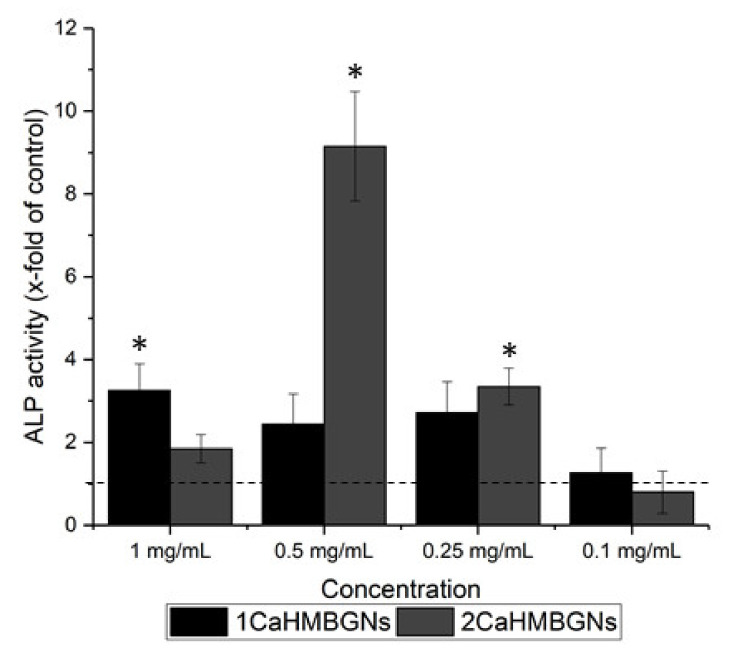
ALP activity of MC3T3-E1 after culture with 1CaHMBGNs and 2CaHMBGNs at different concentrations for 14 days. The ALP activity is presented relative to the control group. Data are presented as mean ± SD and * indicates *p* < 0.05.

We also evaluated the effect of HMBGNs on the osteogenic differentiation of MC3T3-E1. Figure 9 shows the ALP activity (an early indicator of osteogenic differentiation) results of the cells cultured with HMBGNs for 14 days. Compared to the control group, both HMBGNs enhanced the ALP activity at concentrations of 1, 0.5, and 0.25 mg/mL. No significant enhancement in ALP activity could be observed at the concentration of 0.1 mg/mL. It has been known that Ca plays an important role in the regulation of osteoblast differentiation; however, elevated Ca levels could affect cell differentiation negatively. This concentration-dependent effect could explain the osteogenic activity of HMBGNs when cultured with MC3T3-E1 cells at different concentrations. Here the highest ALP activity of 9.15 (±1.32)-fold relative to the control group was observed in 2CaHMBGNs at 0.5 mg/mL concentration, probably due to the concentration of released active ions (Si, Ca) suitable for osteogenic differentiation of MC3T3-E1. The detailed relationship between released ions (profiles, concentrations) from HMBGNs and cells will be investigated in our future study. The present result shows that HMBGNs can enhance the osteogenic differentiation on MC3T3-E1 at suitable concentrations, indicating their potential in bone regeneration applications. However, the concentration of HMBGNs should still be optimized when used for specific drug delivery or tissue engineering applications.

## 4. Conclusions

We demonstrated an effective strategy to synthesize hollow mesoporous bioactive glass nanoparticles (HMBGNs) by combing selective etching and post impregnation. The Stöber method-derived SiO_2_ NPs were used as core templates to assemble mesoporous shells, which ensured the uniform size and shape of HMBGNs. SiO_2_ cores were removed by selective etching with Na_2_CO_3_ solution, while CTAB was decomposed by calcination, leaving mesopores. Ca was incorporated into the particles by post impregnation, which avoided Ca loss during etching of the SiO_2_ core. The doped amount of Ca can be tailored by controlling the concentration of the impregnation solution. HMBGNs were bioactive, as indicated by the rapid formation of HA after immersion in SBF. HMBGNs exhibited concentration-dependent cytotoxicity toward MC3T3-E1 cells and could stimulate osteogenic differentiation at suitable concentrations. The produced HMBGNs show great potential for regenerative medicine applications.

## Data Availability

The data used to support the findings of this study are available from the corresponding author on request.
